# Colonic Gastrointestinal Stromal Tumour Presenting as Intussusception

**Published:** 2013-05-21

**Authors:** Simmi K Ratan, Garima Goel, Parul Sobti, Nita Khurana, Mohit Mathur, Shandip K Sinha, Satish K Aggarwal

**Affiliations:** Department of Pediatric Surgery, Maulana Azad Medical College, New Delhi- India; Department of Pathology, Maulana Azad Medical College, New Delhi- India; Department of Pathology, Maulana Azad Medical College, New Delhi- India; Department of Pathology, Maulana Azad Medical College, New Delhi- India; Department of Pediatric Surgery, Maulana Azad Medical College, New Delhi- India; Department of Pediatric Surgery, Maulana Azad Medical College, New Delhi- India; Department of Pediatric Surgery, Maulana Azad Medical College, New Delhi- India

**Keywords:** Gastrointestinal stromal tumour, Colonic intussusception, Child

## Abstract

Gastrointestinal stromal tumours (GIST) are rare in paediatric patients and have a discrete clinicopathological and molecular divergence from that observed in adults. In the present report we present a case of a 2-month-old female in whom colonic gastrointestinal stromal tumour acted as a lead point of colocolic intussusception. Laparoscopically assisted reduction of the intussusception and resection of tumour was done.

## INTRODUCTION

GIST is the commonest mesenchymal tumour of the gastrointestinal tract in adults, and rarely reported in children [1]. Paediatric GIST are commoner in females, usually arise as multifocal gastric tumours and microscopically show a predominant epithelioid morphology. They are listed in rare lead points of intussusceptions. We present a case of colonic GIST acted as the lead point of colocolic intussusception.

## CASE REPORT

A 2-month-old female child presented with loose stools, bleeding per rectum and excessive crying for 10 days. About 10 hours before presentation, the parents noted mass coming out of rectum. On examination a non reducible bluish-red mass was noticed protruding out of the rectum. On per rectal examination finger could be passed around the mass and it was bleeding on touch. The haematological investigations revealed anaemia with haemoglobin of 9.2gm% however, the biochemical investigations were within normal limits. X-ray and ultrasound of the abdomen showed features suggestive of intussusception. A clinical diagnosis of prolapsing colo-colic intussusception was made and the child was taken up for laparoscopic-assisted surgical correction (Fig. 1). 

Per-operatively a colo-colic intussusception involving the sigmoid colon and prolapsing per rectally was identified. The intussusception was reduced laparoscopically, except for its terminal part. Here the lead point was identified as a 2.5 cm long polypoid mass with irregular surface. Laparoscopic assisted colo-colic resection and anastomosis was done. Other abdominal viscera were normal. Post operative period was uneventful. Gross examination of the resected specimen revealed a polypoid mass of size 2.2 x 2.1.5 cm arising from sigmoid colon, expanding its wall and involving the serosa. Microscopic examination showed a pseudocapsulated transmural tumour with predominant seromuscular involvement. The spindle shaped tumour cells were arranged in sheets and fascicles and had moderate amount of eosinophilic cytoplasm and elongated nuclei. The mitotic rate was 2-3/50 HPF. No pleomorphism and/or foci of necrosis or haemorrhage could be identified throughout the tumour. Sections from the surrounding intestine showed moderate transmural inflammation by mixed inflammatory cells (Fig. 2, 3). On immunohistochemistry, the tumour cells were reactive for vimentin, CD117 (KIT) (Fig. 4); but, were negative for smooth muscle actin (SMA), Factor VIII antigen, neuron specific enolase (NSE) and synaptophysin. In view of the histological and immunohistochemical features, a diagnosis of gastrointestinal stromal tumour was rendered.

**Figure:**
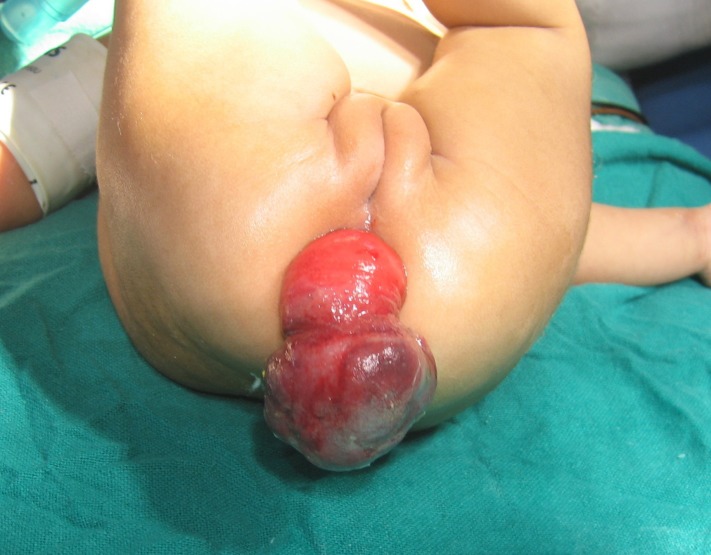
Figure 1: The child with prolapsing intussusception.

**Figure:**
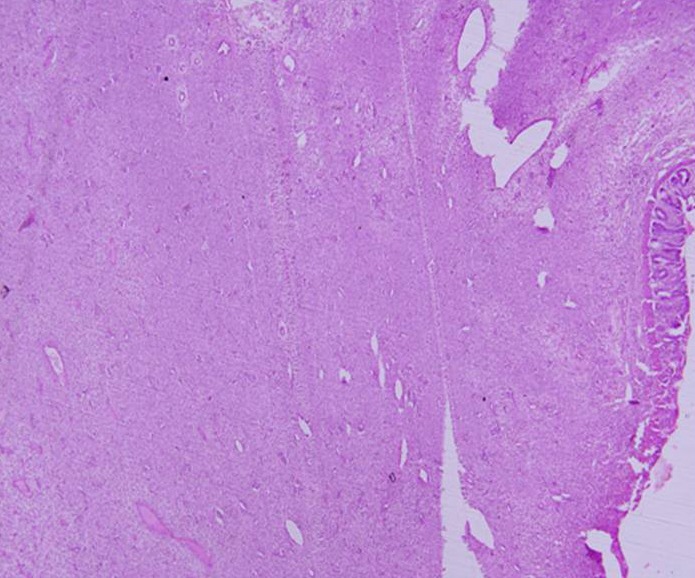
Figure 2: Seromuscular tumour comprised of spindle shaped cells in sheets and fascicles with thinned out overlying mucosa (H/E x 40x).

**Figure:**
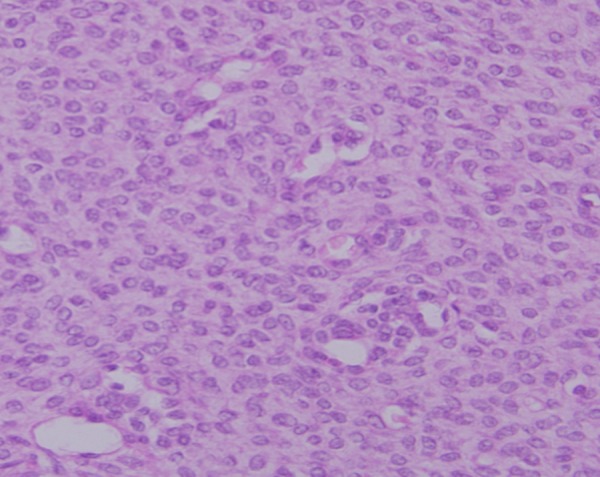
Figure 3: Cellular tumour comprised of monomorphic population of spindle cells with moderate amount of eosinophilic cytoplasm and elongated nuclei. No atypia or mitosis was identified (H/E x 400x).

**Figure:**
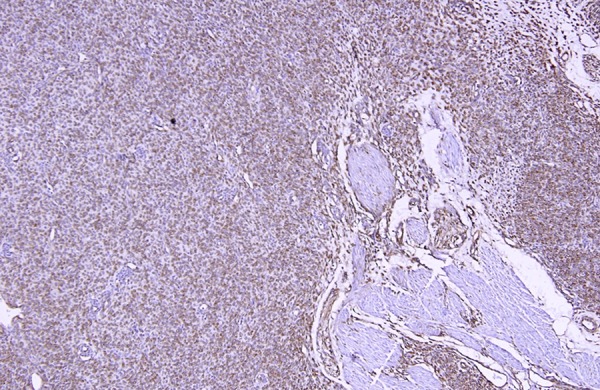
Figure 4: Tumour cells showing strong expression of vimentin.

## DISCUSSION

GIST, a rare tumour of GIT, is actually a sarcoma that is known to arise either from the interstitial cells of Cajal, or their precursor stem cells [1,2]. Sixty to seventy percent cases of this tumour are found to occur in stomach followed by 25-35% in small intestine, rectum ( 5%) and colon (2%) and rarely from mesentry, omentum and peritoneum [1]. Though most GIST grow exophytically, in our patient, the tumour grew intraluminally within the colon which is a rare site for this tumour [2]. 

The commonest presenting symptom in paediatric GIST is anaemia with pallor, fatigue, vertigo, vomiting, abdominal pain, abdominal distension and intestinal obstruction [3]. GIST presenting as intussusception of duodenum, jejunum have also been reported [4]. GIST in paediatric population can arise sporadically, or as a familial disorder (as in Carney-Stratakis syndrome) and even as a component of the non hereditary Carney triad (CT) [5, 6]. Microscopically, paediatric GIST reveals either purely epithelioid or mixed epithelioid/spindle cell morphology, unlike the adult counterparts (predominant spindle cell morphology) [1]. Immunohistochemical analysis of paediatric GIST exhibits positivity with CD117 (KIT-proto-oncogene expressed by interstitial cells and mast cells), and for other markers such as CD34, vimentin and SMA; whereas, these are negative for S-100, desmin, NSE and cytokeratins [3]. Our patient did not exhibit other components of any of the syndromes and her GIST had a predominant spindle cell morphology, unlike that seen in previous reported cases; with positivity for CD117 and vimentin. Kindblom et al, in a series of 78 cases of GIST reported 72% CD34 positivity, and 100% strong, homogeneous immunoreactivity for CD117, a transmembrane tyrosine kinase receptor essential for proper neuronal development [7].

The management of intussusception involves surgical intervention when non operative measures (air reduction, hydrostatic reduction) fail. With the advent of minimally invasive surgery, laparoscopic reduction as a mode of surgical intervention is becoming popular. However limitations in terms of failure of complete reduction or missing a pathologic lead point (PLP) are definitely there as most tactile cues are lost [8]. In our case colocolic intussusceptions, which are known to have least chances of PLP, lead us to offer laparoscopy to this child. 

The preferred treatment modality for paediatric GIST is surgical resection of the tumour along with removal of the involved lymph nodes if present. Though paediatric GIST follows an indolent clinical course and favourable long term prognosis, patients may develop recurrent disease or present with metastasis to the peritoneal cavity, liver and regional lymph nodes at a higher frequency as compared to the adult GIST [9]. Miettinen et al have outlined few tumour related factors (site, size, grade) which help in dictating the prognosis of the GIST, when taken comprehensively into account [10]. Though the risk of prognosis in children is difficult to predict based on the conventional criteria [9]; a low mitotic index, absence of metastasis, complete excision and paediatric age group, dictate good prognosis for her though colonic GIST behave more aggressively than gastric counterparts. On genetic level the prognosis differs in GIST with exon 9 mutation (better) than those with exon 11 [2].

## Footnotes

**Source of Support:** Nil

**Conflict of Interest:** None declared

